# Lung nodule detection using a multi-scale convolutional neural network and global channel spatial attention mechanisms

**DOI:** 10.1038/s41598-025-97187-w

**Published:** 2025-04-10

**Authors:** Yongbin Li, Linhu Hui, Xiaohua Wang, Liping Zou, Stephanie Chua

**Affiliations:** 1 Faculty of Medical Information Engineering, Zunyi Medical University, 563000 Zunyi, Guizhou China; 2 Faculty of Computer Science and Information Technology, Universiti Malaysia Sarawak, 94300 Kota Samarahan, Sarawak Malaysia

**Keywords:** Lung nodule detection, Global channel attention, Channel spatial attention, Multi-scale, Lung cancer, Computed tomography

## Abstract

Early detection of lung nodules is crucial for the prevention and treatment of lung cancer. However, current methods face challenges such as missing small nodules, variations in nodule size, and high false positive rates. To address these challenges, we propose a Global Channel Spatial Attention Mechanism (GCSAM). Building upon it, we develop a Candidate Nodule Detection Network (CNDNet) and a False Positive Reduction Network (FPRNet). CNDNet employs Res2Net as its backbone network to capture multi-scale features of lung nodules, utilizing GCSAM to fuse global contextual information, adaptively adjust feature weights, and refine processing along the spatial dimension. Additionally, we design a Hierarchical Progressive Feature Fusion (HPFF) module to effectively combine deep semantic information with shallow positional information, enabling high-sensitivity detection of nodules of varying sizes. FPRNet significantly reduces the false positive rate by accurately distinguishing true nodules from similar structures. Experimental results on the LUNA16 dataset demonstrate that our method achieves a competitive performance metric (CPM) value of 0.929 and a sensitivity of 0.977 under 2 false positives per scan. Compared to existing methods, our proposed method effectively reduces false positives while maintaining high sensitivity, achieving competitive results.

## Introduction

Lung cancer is one of the most prevalent and deadly forms of cancer, with up to 1.3 million deaths worldwide each year^1^. According to the American Cancer Society’s 2023 statistics, an estimated 238,340 new cases of lung cancer were diagnosed in the United States, with 127,070 deaths attributed to the disease^2^. For cancer, the survival rate of patients largely depends on the stage at which the disease is detected. Statistics show that the five-year average survival rate for lung cancer patients is only about 16%, but early diagnosis and treatment can increase this rate to 70%^3^. In its early stages, lung cancer mainly manifests as lung nodules, and early detection of malignant nodules is the most effective means of preventing lung cancer. Therefore, identifying lung nodules in the early stages of lung cancer is crucial to reducing mortality^4^.

With advancements in medical technology, computed tomography (CT) has become an effective tool for detecting lung nodules, providing a clear and intuitive display of lung lesions and playing a vital role in early lung cancer screening and diagnosis. However, CT scans generate a large volume of slices, leading to a massive volume of data that significantly increases the workload of radiologists and can result in missed detections of small nodules. Furthermore, due to the variability in nodule morphology, nodules often adhere to blood vessels or airway walls, which can easily lead to misdiagnoses^5^. Consequently, the automatic detection of lung nodules using Computer-Aided Detection (CAD) systems has become a major focus in medical image processing research. To assist clinicians in the rapid and accurate diagnosis of lung nodules, various CAD systems have been developed by researchers.

Traditional CAD systems primarily rely on manually designed feature extractors to identify candidate nodules^6–9^. However, these systems often extract only low-level features, such as shape and texture, and are unable to effectively handle the diversity in size, shape, and density of lung nodules, leading to limited generalization capability^10^. With the development of deep learning, convolutional neural network (CNN)-based methods, with their strong capability for self-learning and feature representation, have been widely applied in the field of medical imaging and have achieved outstanding results. In nodule detection, CNN-based methods^11,12^ can automatically learn and extract high-level features of nodules, significantly improving the sensitivity and accuracy of detection. For example, Fu et al.^11^ combined handcrafted and deep features for nodule detection, while Xie et al.^12^ employed a Faster Region Convolutional Neural Network (Faster R-CNN)^13^ architecture with two region proposal networks (RPNs) and a deconvolution layer to integrate shallow information and generate more candidate regions for lung nodule detection. However, these methods are typically based on 2D CNNs and cannot fully utilize the spatial information in 3D CT images. Therefore, recent research has increasingly focused on 3D convolutional neural networks (3D CNNs)^10,14–17^. For example, Ding et al.^16^ proposed a Faster R-CNN network based on a deconvolution structure to detect suspicious nodules layer by layer, followed by a deep 3D CNN network to reduce false positives. Cao et al.^17^ developed a two-stage convolutional neural network (TSCNN) model for lung nodule detection, which first uses a U-Net based on ResDense^18^ for nodule detection and then reduces false positives using an ensemble learning framework based on 3D CNNs.

Due to the significant heterogeneity of lung nodules in terms of size, shape, and density, a single feature extraction method struggles to comprehensively capture multidimensional structural information, thereby limiting the generalization capability of models. To address this issue, researchers have proposed multi-scale feature extraction methods to capture both local and global information at different scales. For example, Tang et al.^19^ proposed a multi-scale feature 3D U-Net^20^ based on transfer learning; Zheng et al.^21^ developed a multi-scale feature detection network; and Zhao et al.^22^ combined multi-scale features with Faster R-CNN to improve the detection of small-sized lung nodules. Attention mechanisms, with their advantages in feature selection, have been introduced into lung nodule detection. By dynamically adjusting feature weights, they can adaptively highlight important features related to nodule detection while suppressing irrelevant ones, providing new insights for lung nodule detection^23–28^. For instance, Li et al.^23^ incorporated the Squeeze-and-Excitation Network (SE-Net)^29^ into residual blocks^30^ to enhance detection performance; Zhao et al.^25^ proposed a 3D CNN model based on channel and spatial attention within a multi-scale architecture, combined with a Feature Pyramid Network (FPN)^31^ for two-stage lung nodule detection; Cao et al.^26^ combined FPN with attention modules to improve the predictive ability for small-sized nodules.

Despite the significant progress achieved by CNN-based automatic lung nodule detection systems, several challenges remain. On one hand, small-sized lung nodules are difficult to detect due to their small volume and low contrast with surrounding tissues, making it challenging to effectively extract their texture features, which often leads to missed detections. On the other hand, the morphological diversity of lung nodules and their high similarity to normal anatomical structures (such as blood vessels and airway walls) further increase the risk of misdiagnosis, resulting in a higher false-positive rate in detection systems.

To address the aforementioned challenges, this study proposes a Global Channel Spatial Attention Mechanism (GCSAM), which introduces global contextual information to adaptively adjust feature weights, particularly optimizing the representation of critical features for small-sized and complex-shaped nodules. Based on this mechanism, the Res2GCSA module is proposed by integrating Res2Net^32^ with GCSAM. This module combines multi-scale feature extraction with global contextual attention, significantly enhancing the network’s ability to represent features of small-sized and complex-shaped lung nodules.

Based on GCSAM, this study constructs a two-stage automatic lung nodule detection system comprising a Candidate Nodule Detection Network (CNDNet) and a False Positive Reduction Network (FPRNet). CNDNet leverages the Res2GCSA module to adaptively adjust feature weights across different scales, achieving efficient multi-scale feature fusion. Inspired by the approach in^33^, a Hierarchical Progressive Feature Fusion (HPFF) method is designed to progressively integrate shallow positional information with deep semantic information. FPRNet, on the other hand, employs the Res2GCSA module to build a feature encoder, refining feature representations by combining multi-scale features with global contextual information, thereby enhancing the distinction between true nodules and similar structures.

Experimental validation on the LUNA16 dataset demonstrates that the proposed method achieves a competitive performance metric (CPM) value of 0.929 and a sensitivity of 0.977 at two false positives per scan (FPs/scan), outperforming most existing methods. The method effectively reduces false positives while maintaining high sensitivity, showcasing its practicality and potential in lung nodule detection.

This paper is organized as follows: “Methods” introduces the proposed method; “Experiment and results” describes the datasets, preprocessing methods, training details, and evaluation metrics, along with the experimental results; “Discussion” provides an in-depth discussion of the experimental results, including comparative analysis, ablation studies, and performance in detecting nodules of different sizes and morphologies; and “Conclusions” concludes the paper with the main findings and future research directions.

## Methods

**Fig. 1:**
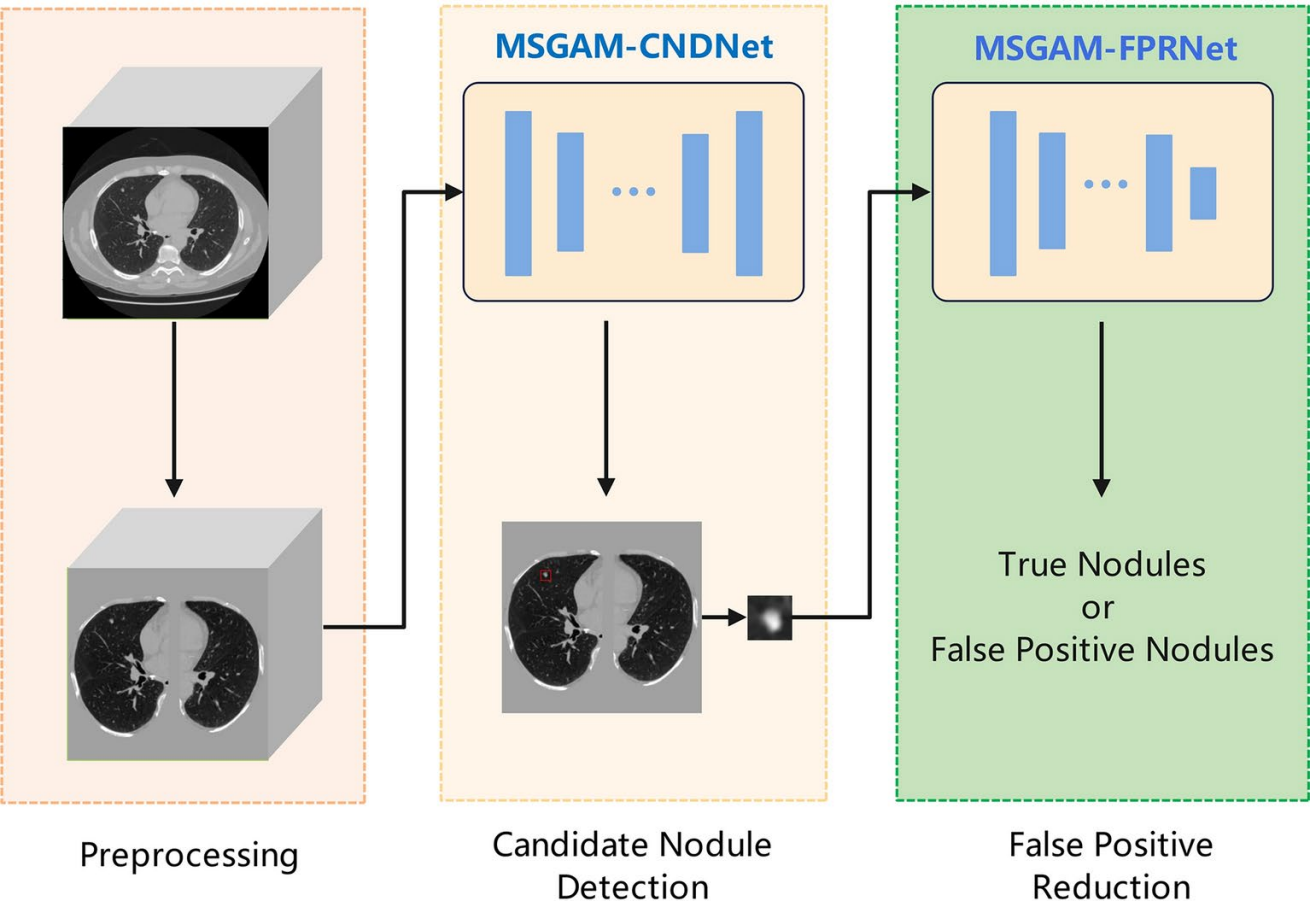
The overall workflow of the lung nodule detection system.

The overall workflow of the proposed lung nodule detection system is illustrated in Fig. 1. Firstly, in the preprocessing stage, the original CT images undergo denoising and enhancement operations. The preprocessed image patches are fed into the CNDNet to detect all candidate nodules. Finally, the detected candidate nodules are input into the FPRNet to classify the nodules, effectively distinguishing true nodules from false positives, thereby further enhancing the accuracy and robustness of the detection.

### Candidate nodule detection network

**Fig. 2:**
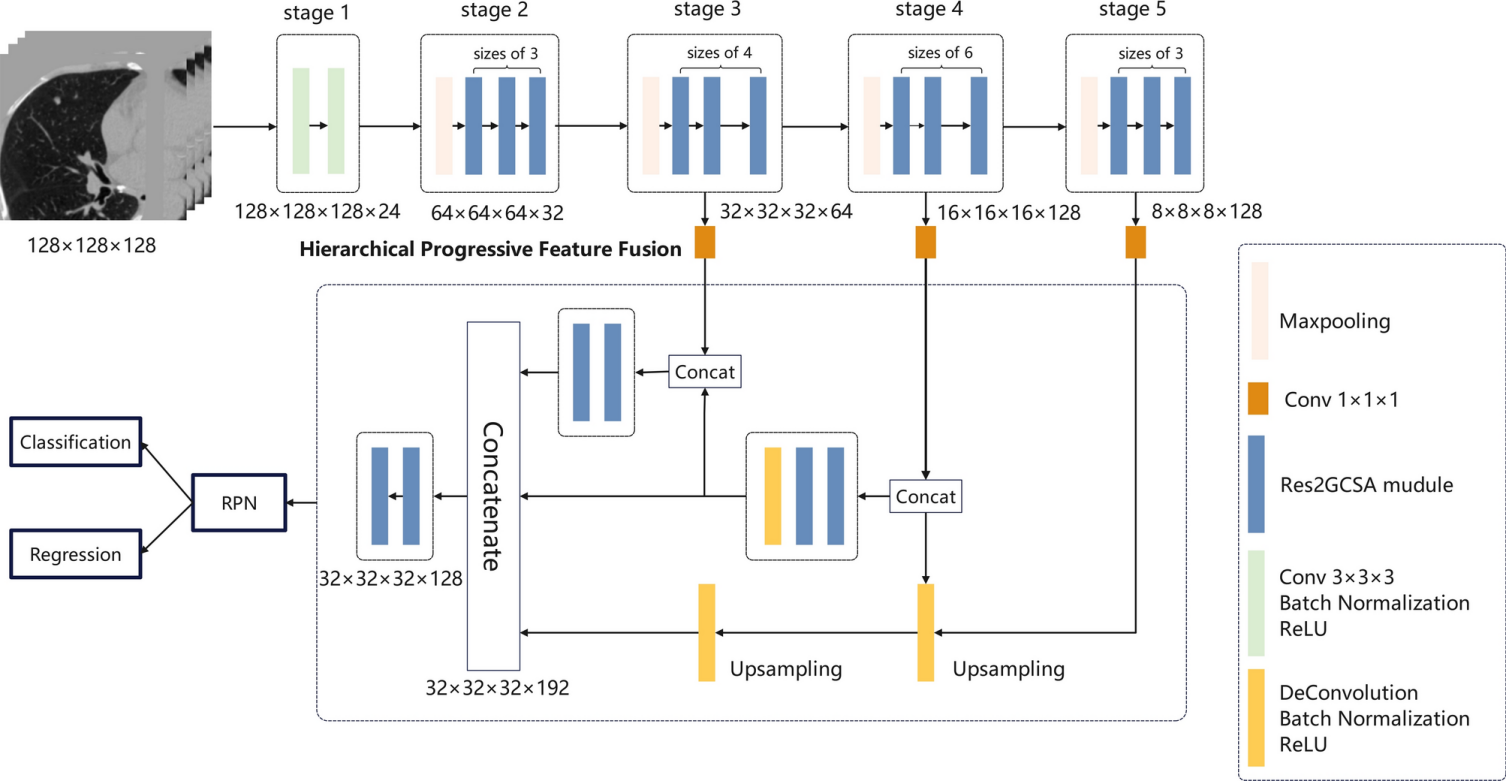
The architecture of candidate nodule detection network.

The architecture for the proposed 3D CNDNet featuring a multi-scale feature extraction and fusion mechanism is illustrated in Fig. 2. The Res2GCSA module was constructed as the fundamental unit of the network, by integrating the Global Channel Spatial Attention Mechanism (GCSAM) with Res2Net^32^. This module not only captured local features of nodules across multiple scales but also utilizes GCSAM to adaptively adjust feature weights, thereby focusing on critical regions. Subsequently, the HPFF method was employed to fuse features at different scales, enhancing the network’s capability to detect lung nodules of varying sizes and complex shapes. Finally, the network used the 3D Region Proposal Network (RPN)^13^ for candidate region generation and precise detection of lung nodules.

The input to CNDNet was a 3D image block of size 128 × 128 × 128, processed through five progressively deeper feature extraction stages. In the first stage, the input features passed through a standard convolutional layer to perform initial processing, extracting the structural information and low-level features from the raw image. Subsequently, in each stage, the feature maps were processed by multiple Res2GCSA modules to obtain deeper feature representations. At the end of each stage, a MaxPooling operation with a stride of 2 was introduced to reduce computational complexity while further extracting higher-level semantic information, providing a rich feature foundation for subsequent candidate region generation and detection.

In the network, shallow features are more specific and contain more positional information, which is crucial for the prediction of small-sized targets. However, as the network depth increases, the feature representations become more abstract, encompassing richer semantic information, but potentially losing positional information, which is detrimental to the detection of small nodules. To balance this issue, this study introduced the HPFF method to gradually integrate shallow and deep features. HPFF first used deconvolution layers to progressively up-sample deep features, restoring their spatial resolution and incorporating Batch Normalization (BN) to stabilize the training process. The feature maps from the previous stage were concatenated with the up-sampled features after passing through a 1 × 1 × 1 convolution. This fusion ensured the integrity of information while enhancing the richness of feature representation^33^. Subsequently, the fused features were processed again through the Res2GCSA module to optimize feature expression and selection. Finally, the feature maps from all levels were unified to the same size (32 × 32 × 32) and concatenated along the channel dimension to form a high-dimensional feature map with 192 channels. This high-dimensional feature map underwent further fusion and refinement through an additional Res2GCSA module.

The fused features were then fed into the classification and regression modules of the RPN^13^ for target classification and bounding box localization. To ensure sensitivity to small nodules and nodules of varying sizes, three different anchor box sizes of 5, 10, and 20 were adopted. Each anchor box consisted of a confidence score *p*, representing the predicted probability of nodule, and four regression terms, which were used to predict the nodule’s centre coordinates (x, y, z) in three-dimensional space and its diameter.

### Res2GCSA module

**Fig. 3:**
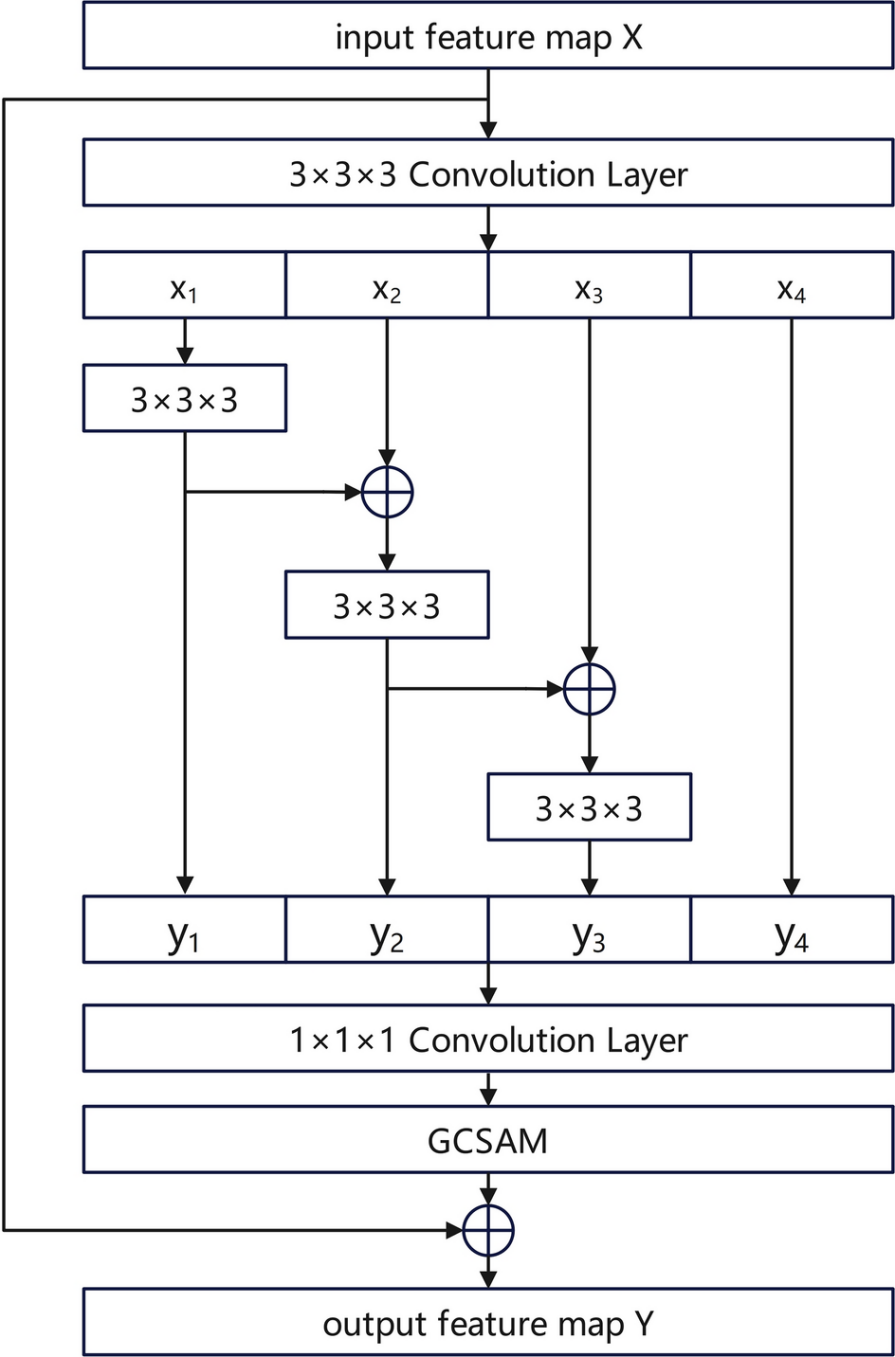
The structure of Res2GCSA module.

The proposed Res2GCSA module was constructed based on the Res2Net block^32^ with the introduction of GCSAM. This module combined the multi-scale feature extraction capabilities of Res2Net with global channel-spatial attention to enhance the module’s performance in detecting lung nodules of varying sizes and complex shapes, as illustrated in Fig. 3. The Res2GCSA module first used a 3 × 3 × 3 convolution to extract features from the input feature map X. The output feature map was then evenly divided into four subsets along the channel dimension, denoted as ${x}_{1}$, ${x}_{2}$, ${x}_{3}$, ${x}_{4}$. Each feature subset ${\text{x}}_{\text{i}}$ was added to the output ${y}_{i-1}$ of the previous convolution layer ${K}_{i-1}$, and the result was fed into the convolution layer ${K}_{i}$ within the same path to obtain the feature subset ${y}_{i}$. The process is formulated as shown in Eq. (1):

1 $${y}_{i}=\left\{\begin{array}{c}{K}_{i}{x}_{i}, i=1;\\ {K}_{i}\left({y}_{i-1}+{x}_{i}\right), 2\le i\le 3;\\ {x}_{i}, i=4\end{array}\right.$$

In the fourth path, no operations were performed to facilitate feature reuse. The first three paths used three 3 × 3 × 3 convolutional operations to increase the receptive field of each feature subset, allowing the network to introduce more non-linear representation capabilities in different paths while ensuring that multi-scale features were effectively extracted in each path.

Subsequently, all feature subsets [${y}_{1}$, ${y}_{2}$, ${y}_{3}$, ${y}_{4}$] were concatenated along the channel dimension to enhance the diversity and expressive power of the features. The concatenated output was then processed by a 1 × 1 × 1 convolution operation to restore the original number of channels, followed by BN to ensure training stability. To further enhance the model’s expressive capability through attention mechanisms, the GCSAM was introduced at the end of the module, adaptively adjusting feature weights based on global contextual information and spatial attention. Finally, the extracted features were combined with the shortcut connection using element-wise addition and passed through a ReLU activation function, enabling the network to maintain deeper feature representation across multiple scales.

### Global channel spatial attention mechanism

**Fig. 4:**
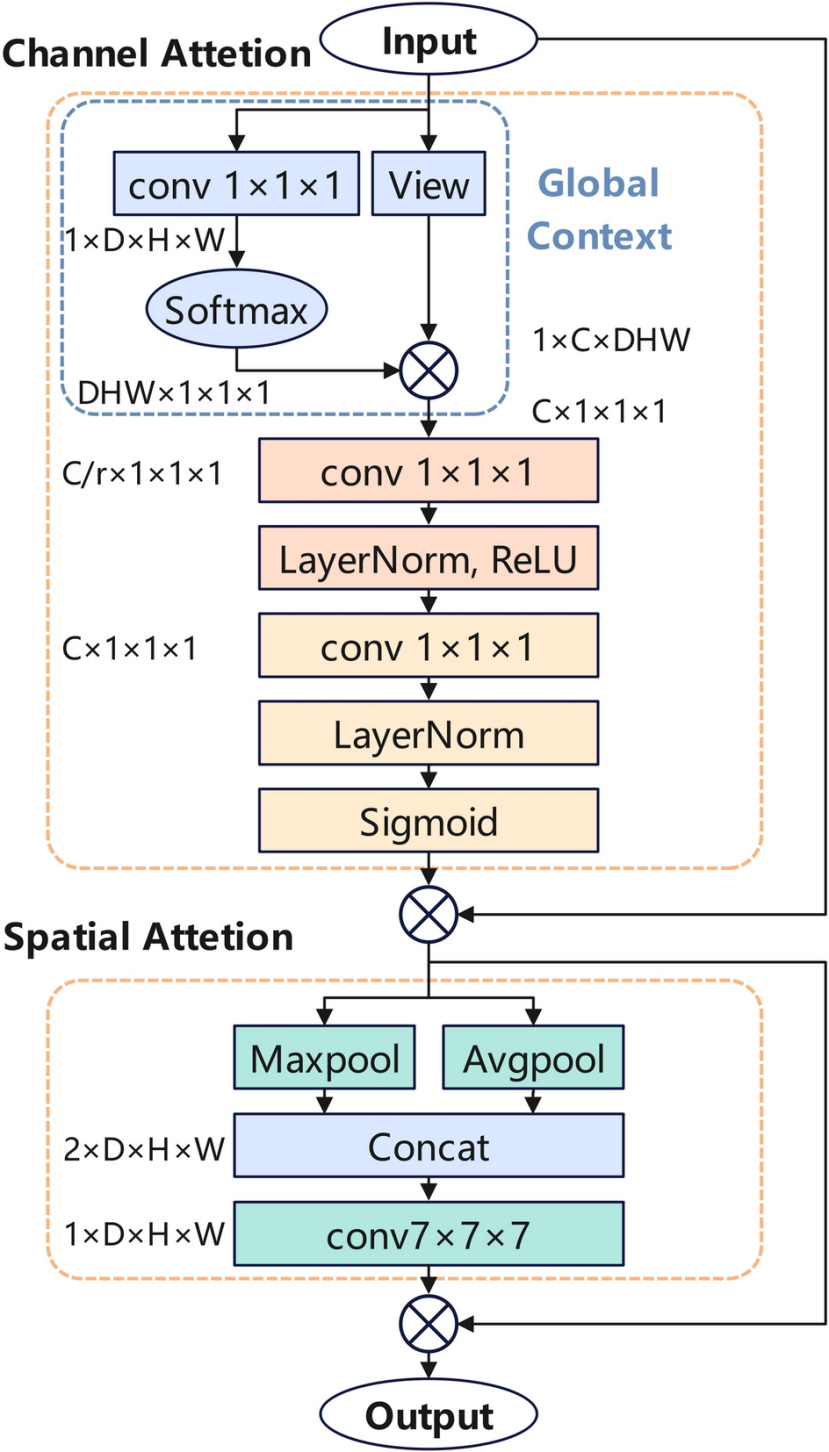
The structure of GCSAM module.

This paper presents a novel GCSAM module, as shown in Fig. 4. The GCSAM module consisted of two main components: channel attention and spatial attention. The channel attention integrated global contextual information at the channel level^29,34^, which helped to better identify and highlight subtle features relevant to nodule detection. The spatial attention further refined the features to enhance accurate localization capability^35^. Through this dual focus, GCSAM effectively improved the model’s generalization ability across multiple scales and complex scenarios.

The channel attention module aimed to adaptively adjust the feature weights of each channel using global contextual information. To achieve this, a Global Context (GC) module^34^ was introduced to capture and integrate global contextual information. This module was essentially a simplified version of the Non-local (NL) block^36^. The GC module first performed global attention pooling in the spatial domain to compute a global context vector, which captured the overall information of the input feature map and weighted the features at each position accordingly. Specifically, given an input feature map $\text{X}\in {R}^{C\times D\times H\times W}$, where C represents the number of channels of the 3D image, and D, H, and W represent the depth, height, and width, respectively, a 1 × 1 × 1 convolutional layer was applied to generate a global context vector. This vector was then multiplied by the reshaped feature map X to obtain the global context weights:

2 $$G=X\bullet Softmax({Conv}_{1\times 1\times 1}\left(X\right))$$

Next, the global context weights were passed through a feature transformation module composed of two 1 × 1 × 1 convolutional layers. The channel dimension C was first reduced to C/r, where r was the channel reduction ratio. Layer normalization (LayerNorm) and a ReLU activation function were then applied sequentially, followed by another convolutional layer to restore the original channel dimension. Compared to SE-Net^29^, a LayerNorm was added after each convolutional layer to further stabilize the feature distribution and improve the model’s generalization ability. Finally, the attention weights were generated through a Sigmoid function as shown in Eq. (3):

3 $${M}_{c}=\sigma (LayerNorm\left({Conv}_{1\times 1\times 1}\left(ReLU\left(LayerNorm\left({Conv}_{1\times 1\times 1}\left(G\right)\right)\right)\right)\right))$$

where σ(⋅) denotes the Sigmoid activation function. Unlike traditional addition operations^34^, element-wise multiplication was used here to apply the computed global context weights to the multi-scale feature maps, resulting in the final output feature map ${X}{\prime}$ as shown in Eq. (4)

4 $${X}{\prime}=X \odot {M}_{c}$$

The spatial attention module further refined the feature maps adjusted by the channel attention module along the spatial dimensions to enhance localization accuracy. It first generated two spatial feature maps through max pooling and average pooling operations, which were then concatenated along the channel dimension to form a fused spatial feature map, *X*_*c*_ as shown in Eq. (5):

5 $${X}_{c}=Concat(AvgPool\left({X}{\prime}\right),MaxPool\left({X}{\prime}\right))$$

The concatenated feature map was then passed through a 7 × 7 × 7 convolution operation to capture more extensive global information in the spatial domain, resulting in the final spatial attention weights ${M}_{s}$as shown in Eq. (6):

6 $${M}_{s}=\sigma ({Conv}_{7\times 7\times 7}\left({X}_{c}\right))$$

Finally, the spatial attention weights ${M}_{s}$ were element-wise multiplied with the channel attention-adjusted feature map ${X}{\prime}$ to obtain the final output feature map $Y$ as shown in Eq. (7):

7 $$Y={X}^{{^{\prime}}} \odot {M}_{s}$$

### Loss function

The loss function that was used comprised two components: the classification loss ${L}_{cls}$ and the regression loss ${L}_{reg}$. For the classification loss ${L}_{cls}$, the binary cross-entropy (BCE) loss function was used, while the regression loss ${L}_{reg}$ was defined by the smooth L1 loss function^12^. The Intersection over Union (IoU) between the anchor box and the ground truth bounding box was used to determine whether a candidate region was a positive sample for nodule detection. An IoU greater than 0.5 was considered a positive sample, whereas an IoU less than 0.02 with all ground truth boxes was considered a negative sample; all other cases were ignored. The multi-task loss function for anchor $i$ is defined as shown in Eq. (8):

8 $$L\left({p}_{i},{t}_{i}\right)={L}_{cls}\left({p}_{i},{p}_{i}^{*}\right)+{p}_{i}^{*}{L}_{reg}\left({t}_{i},{t}_{i}^{*}\right)$$

where ${p}_{i}$ is the predicted probability that anchor iii contains a nodule, and ${p}_{i}^{*}$ is the ground truth label. Notably, the regression loss was computed only for positive samples.

For the classification loss ${L}_{cls}$, since it was a binary classification problem using a sigmoid activation function, the BCE loss was used, as defined in Eq. (9):

9 $${L}_{cls}\left({p}_{i},{p}_{i}^{*}\right)=-[{p}_{i}^{*}\cdot \text{log}\left({p}_{i}\right)+(1-{p}_{i}^{*})\cdot \text{log}\left(1-{p}_{i}\right)$$

For the regression loss ${L}_{reg}$, the smooth L1 loss was used. Compared to the L2 loss function, the smooth L1 loss better balanced errors, avoiding excessive sensitivity to single samples, and addressing the issue of non-differentiability at zero in the L1 norm loss. The regression loss is defined as shown in Eq. (10):

10 $${L}_{reg}\left({t}_{i},{t}_{i}^{*}\right)=\left\{\begin{array}{c}0.5{\cdot ({t}_{i}-{t}_{i}^{*})}^{2} if |{t}_{i}-{t}_{i}^{*}|<1\\ \left|{t}_{i}-{t}_{i}^{*}\right|-0.5 otherwise\end{array}\right.$$

where ${t}_{i}$ and ${t}_{i}^{*}$ are the relative representations of the predicted and ground truth nodule coordinates and diameters, respectively. Specifically, ${t}_{i}$ was defined as shown in Eq. (11):

11 $${t}_{i}=\left(\frac{x-{x}_{a}}{{d}_{a}},\frac{y-{y}_{a}}{{d}_{a}},\frac{z-{z}_{a}}{{d}_{a}},\text{log}(\frac{d}{{d}_{a}})\right)$$

where $\left(x,y,z,d\right)$ are the predicted coordinates and diameter of the nodule in the original image, and $({x}_{a},{y}_{a},{z}_{a},{d}_{a})$ are the coordinates and size of anchor $i$. The ground truth nodule coordinates ${t}_{i}^{*}$ were defined as shown in Eq. (12):

12 $${t}_{i}^{*}=\left(\frac{{x}^{*}-{x}_{a}}{{d}_{a}},\frac{{y}^{*}-{y}_{a}}{{d}_{a}},\frac{{z}^{*}-{z}_{a}}{{d}_{a}},\text{log}(\frac{{d}^{*}}{{d}_{a}})\right)$$

where $({x}^{*},{y}^{*},{z}^{*},{d}^{*})$ represent the ground truth nodule coordinates and diameter.

### False positive reduction network

**Fig. 5:**
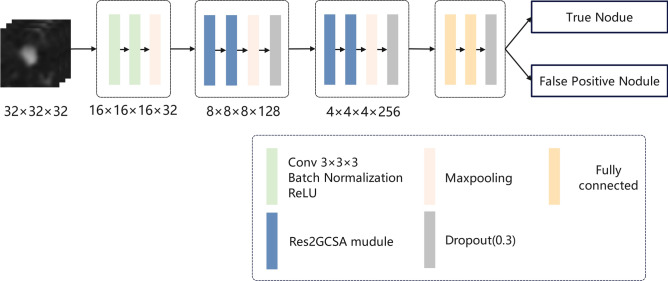
The architecture of false positive reduction network.

After the first stage of candidate nodule detection, a large number of false-positive nodules were typically generated. To identify true positive nodules from these numerous candidates, an FPRNet for false-positive reduction was designed based on the Res2GCSA module, as illustrated in Fig. 5.

FPRNet consisted of a preprocessing block, a feature encoder, and two fully connected layers. To avoid potential biases introduced by the detection model, features extracted by the detection model were not directly used as input^21^. Instead, 32 × 32 × 32 image patches were cropped from the original CT images based on the coordinates of the candidate nodules, allowing feature learning to be independent of the detection model. This approach focused more on distinguishing true nodules from false positives. In the encoder section, the image patches underwent feature extraction and down-sampling through multiple layers of the Res2GCSA module to obtain high-dimensional deep feature representations. Dropout layers^37^ were added after each layer to prevent overfitting and enhance the model’s generalization capability. Finally, the feature maps output from the encoder were flattened into a one-dimensional vector and passed through two fully connected layers to generate classification probabilities for true and false-positive nodules, optimized using a binary cross-entropy loss function.

### Code availability

The custom code used for this study is available in the Zenodo repository (10.5281/zenodo.14986604) for reference. It is freely accessible for academic research and non-commercial purposes.

## Experiment and results

### Dataset

This study used the publicly available LUng Nodule Analysis 2016 challenge (LUNA16) dataset^38^. The LUNA16 dataset was constructed by Setio et al. based on the Lung Image Database Consortium and Image Database Resource Initiative (LIDC-IDRI)^39^ and was used for the objective evaluation of automated lung nodule detection systems. The LIDC-IDRI database included 1018 cases of CT image data, with each case containing between 200 to 500 CT images. These medical images were stored in Digital Imaging and Communications in Medicine (DICOM) format and were annotated by four radiologists in two phases. Due to the significant variability in the LIDC-IDRI data, the LUNA16 dataset was curated by excluding CT scans with slice thickness greater than 2.5 mm. Additionally, to ensure the reliability of the annotations, LUNA16 only retained nodules larger than 3 mm in diameter that were jointly annotated by three to four experts. In the end, LUNA16 selected 888 CT scans and 1186 nodules with annotation information as the experimental data, with an average diameter of 8.3 mm. For the false-positive reduction challenge, the dataset also included 754,975 candidate nodules, out of which 1557 were confirmed as true nodules.

### Preprocessing

**Fig. 6:**
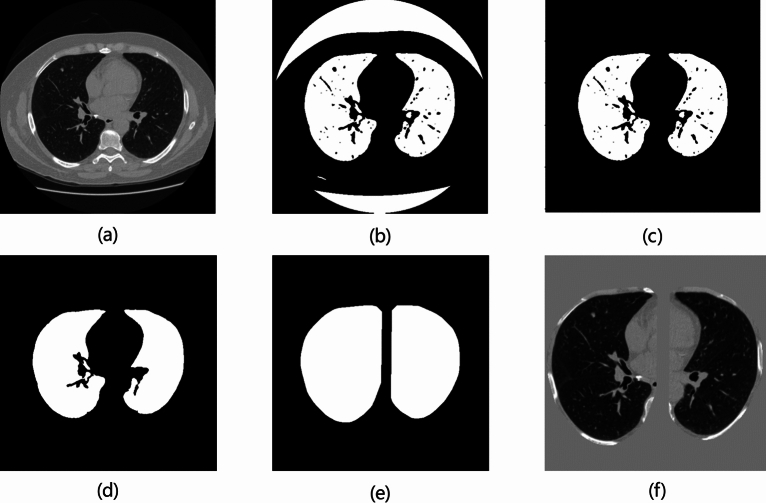
Preprocessing for lung nodule detection.

The original lung CT images contain a large amount of irrelevant information, such as bones, airways, blood vessels, and other non-pulmonary parenchymal tissues. To eliminate these interferences, we applied a preprocessing approach referencing the methods in^15,21^, as outlined in Fig. 6.

The first step involved extracting a lung mask^40^. The raw CT images (Fig. 6a) are imported and thresholded at − 600 Hounsfield Units (HU) to create an initial binary mask (Fig. 6b). Next, a connected component analysis was performed on the mask to remove non-pulmonary parenchymal regions connected to the background (Fig. 6c). Then, an erosion operation was applied to remove isolated noise points, followed by a hole-filling algorithm to fill gaps within the mask (Fig. 6d). To ensure the completeness and continuity of the lung region, the segmented area underwent convex hull processing to fill small gaps caused by imperfect segmentation or noise. A binary dilation method was iteratively applied 10 times to generate a complete lung parenchymal mask (Fig. 6e).

Considering that the HU values of lung tissues were around − 500, only regions within the HU range of [− 1200, 600] were retained and normalized to a grayscale range of [0, 255]. The normalized image was then multiplied by the lung parenchymal mask to remove non-lung regions. The remaining background noise and unrelated tissues (such as bones and blood vessels) were filled with a value of 170^21^. The processed image is shown in Fig. 6f.

### Experimental setup

The experimental environment used the Windows 11 operating system, with hardware configurations including an Intel Core i7-12700 K CPU at 3.6 GHz and 32 GB of memory. All network models were implemented in Python 3.8.8 and trained using the PyTorch 1.8.1 deep learning framework on an NVIDIA GeForce RTX 3090 GPU with 24 GB of memory. Both CNDNet and FPRNet were optimized using Stochastic Gradient Descent (SGD) with an initial learning rate of 0.01, a momentum of 0.9, and a weight decay factor of 0.0001. The model training employed ten-fold cross-validation, where 9 subsets were used as the training set, and the remaining 1 subset was used as the test set. The final results were obtained by combining the predictions from all 10 subsets.

During the nodule detection phase, the preprocessed CT images were augmented using random flipping, rotation, and cropping techniques to enhance the positive samples. The total number of training epochs was set to 200; the learning rate was reduced to 0.001 at the 50th epoch and further decreased to 0.0001 after the 100th epoch, with a batch size of 4. In the testing phase, the preprocessed CT images were divided into image blocks of size 208 × 208 × 208 for prediction. The prediction results of all image blocks were merged using Non-Maximum Suppression (NMS)^41^ with an IoU threshold of 0.05 to consolidate overlapping candidate results, yielding the final detection outcomes.

During the false-positive reduction phase, due to the highly imbalanced training data, data augmentation was employed to expand the 1557 positive samples to 31,140, while only approximately 3% of the negative samples, 22,650 samples, were randomly selected and retained. The total number of training epochs was 50, with the learning rate reduced to 0.001 at the 15th epoch and further to 0.0001 at the 30th epoch, using a batch size of 8.

### Evaluation metrics

In medical image analysis, particularly in lung nodule detection tasks, the commonly used evaluation standard is the Free-Response Receiver Operating Characteristic (FROC) curve^42^. The FROC curve is used to evaluate the sensitivity of a detection model concerning the average number of false positives per scan (FPs/scan), thereby assessing the model’s performance across different thresholds. The closer the curve is to the upper left corner, the better the detection performance. Specifically, true positives (TP) refer to instances where both the annotation and detection are identified as nodules. If the detected nodule’s center is within the radius of the annotated nodule, it is considered a TP; otherwise, it is deemed a false positive (FP). False negatives (FN) are cases where a nodule is annotated but not detected. The fewer the false negatives, the higher the model’s sensitivity and the lower the miss rate. The formula for sensitivity is as shown in Eq. (13):

13 $$Sensitivity=TP/(TP+FN)$$

To compare different methods, this study adopts the Competition Performance Metric (CPM)^43^ as the primary evaluation metric, calculated on the LUNA16 dataset to compare the detection effectiveness of various models. In lung nodule detection tasks, CPM is widely used as a key indicator of overall model performance. CPM is defined as the average sensitivity for FPs/scan values of 0.125, 0.25, 0.5, 1, 2, 4, and 8. Compared to a single sensitivity or false positive rate, CPM provides a more comprehensive reflection of the model’s performance under different detection conditions. A higher CPM score indicates better model performance. The formula for CPM is as shown in Eq. (14):

14 $$CPM=\frac{1}{7}\sum_{i=\{{0.125,0.25,0.5,1},{2,4},8\}}{Sensitivity}_{fpr=i}$$

where $fpr$ represents the FPs/scan, and ${Sensitivity}_{fpr=i}$ denotes the sensitivity at a given $fpr=i$.

## Experimental results

**Fig. 7:**
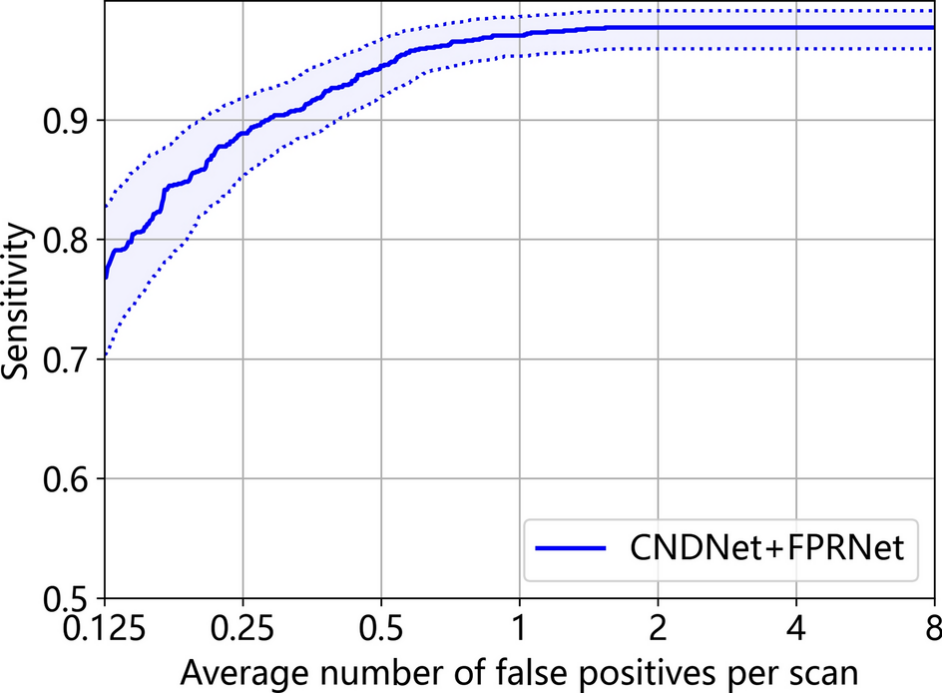
FROC curve of proposed method. The blue solid line illustrates the sensitivity across varying FPs/scan, while the blue dashed lines represent the 95% confidence interval boundaries.

In this section, the performance of the proposed method on the LUNA16 dataset for lung nodule detection is presented. Figure 7 shows the FROC curve of proposed method (CNDNet + FPRNet). The curve indicated that the sensitivity reached 0.977 at 2, 4, and 8 FPs/scan. To comprehensively evaluate the overall performance, the CPM value was calculated. The results showed that the proposed method achieved a CPM of 0.929, validating the effectiveness of the multi-scale feature extraction and the global channel spatial attention mechanism. This outcome suggested that the proposed model could maintain high detection sensitivity while significantly reducing false positive rates, highlighting its potential utility in early lung nodule detection.

A detailed analysis of the performance comparison between CNDNet and CNDNet + FPRNet on the LUNA16 dataset was conducted, as shown in Table 1. It was evident that the introduction of FPRNet led to an improvement in sensitivity across all FPs/scan. Specifically, when FPs/scan was 0.125 and 0.25, the sensitivity of CNDNet + FPRNet increased from 0.704 to 0.773 and from 0.845 to 0.888, respectively. This indicated that incorporating FPRNet significantly reduced the false negative rate, particularly under low false-positive conditions. Overall, the CPM improved from 0.902 to 0.929, further confirming the critical role of FPRNet in improving overall detection performance. FPRNet leveraged the GCSAM to refine feature extraction, especially by utilizing global contextual information to suppress irrelevant features, thereby effectively improving the sensitivity to true nodules and comprehensive detection performance.

**Table 1:** Performance comparison with CNDNet and CNDNet + FPRNet.

Methods	Sensitivity of different FPs/scan							CPM
	0.125	0.25	0.5	1	2	4	8	
CNDNet	0.704	0.845	0.933	0.968	0.975	0.976	0.976	0.911
CNDNet + FPRNet	0.773	0.888	0.945	0.971	0.977	0.977	0.977	0.929

## Discussion

### Performance comparison

The performance comparison between proposed method and previous research on the LUNA16 dataset is shown in Table 2. The results of the comparison methods were cited from the original publications and were evaluated on the same LUNA16 dataset to ensure comparability.

**Table 2:** Performance comparison with the prior methods.

Methods	Sensitivity of different FPs/scan							CPM	p-value
	0.125	0.25	0.5	1	2	4	8		
Zhao et al.^22^	0.677	0.741	0.816	0.85	0.89	0.905	0.925	0.829	< 0.05
Zhu et al.^15^	0.677	0.737	0.815	0.848	0.879	0.907	0.922	0.842	< 0.05
Li et al.^23^	0.739	0.803	0.858	0.888	0.907	0.916	0.920	0.862	< 0.05
Mei et al.^10^	0.712	0.802	0.865	0.901	0.937	0.946	0.955	0.874	< 0.05
Ding et al.^16^	0.748	0.853	0.887	0.922	0.938	0.944	0.946	0.891	< 0.05
Khosravan et al.^14^	0.709	0.836	0.921	0.953	0.953	0.953	0.953	0.897	< 0.05
Zheng et al.^21^	0.770	0.849	0.904	0.940	0.957	0.959	0.959	0.906	< 0.05
Song et al.^46^	0.702	0.826	0.914	0.959	**0.978**	**0.979**	**0.980**	0.906	< 0.05
Mkindu et al.^27^	*	*	*	0.944	*	0.975	*	0.911	< 0.05
Cao et al.^17^	0.846	**0.899**	0.925	0.936	0.949	0.957	0.960	0.925	0.429
Zhang et al.^28^	**0.856**	0.898	0.930	0.945	0.953	0.962	0.962	0.927	0.916
proposed method	0.773	0.888	**0.945**	**0.971**	0.977	0.977	0.977	**0.929**	N/A

As shown in Table 2, the proposed method achieved a CPM value of 0.929, demonstrating competitive detection performance compared to previous studies. However, other methods not listed in the table have been reported in the literature. For instance, Zhang et al.^44^ reported a CPM of 0.941 using a 3D residual U-Net for lung nodule segmentation, which significantly differs from the RPN-based detection framework employed in this study. Additionally, Gu et al.^45^ proposed a detection method incorporating a vessel suppression model, achieving a CPM value of 0.977 and sensitivity of 0.986 at 8 FPs/scan. However, their study utilized an additional private dataset for model training and preprocessing, differing from this study’s approach of relying solely on the LUNA16 dataset. As a result, these methods were not included in the comparison.

Further analysis of the results in Table 2 reveals that our proposed method demonstrates high sensitivity at FPs/scan values of 0.5 and 1, achieving 0.945 and 0.971, respectively, surpassing other methods listed in the table. However, at 2, 4, and 8 FPs/scan, the multi-scale 3D anchor-free deep learning network proposed by Song et al.^46^ exhibits higher sensitivity, reaching 0.978, 0.979, and 0.980, slightly exceeding the proposed method’s 0.977. Nonetheless, our proposed method achieves a CPM value of 0.929, which is higher than that of Song et al.^46^ 0.906. This indicates that the proposed method effectively balances detection performance at low FPs/scan with overall detection capability.

To validate the statistical significance of the performance differences between the proposed method and the methods reported in the literature, a one-sample t-test^47^ was conducted based on the CPM values obtained from tenfold cross-validation. The CPM value of each method was used as the assumed population mean. The results indicate that the performance differences between the proposed method and most of the compared methods were statistically significant (p < 0.05). However, comparisons with Cao et al.^17^ and Zhang et al.^28^ yielded p-values of 0.429 and 0.916, respectively, indicating no statistically significant performance differences. These results confirm that the proposed method performs comparably to existing approaches, with statistical analysis indicating its effectiveness in lung nodule detection.

### Ablation experiment

In this section, a series of ablation experiments were conducted to analyze the impact of each proposed module on lung nodule detection performance. Eight different experimental configurations were designed, all adopting a two-stage detection framework that included candidate nodule detection and false positive reduction. Table 3 summarizes the sensitivity at 8 FPs/scan along with its 95% confidence interval, as well as the overall performance metric CPM.

**Table 3:** Performance comparison of different methods.

Methods	Sensitivity [lower bound, upper bound]	CPM [lower bound, upper bound]
ResNet	0.923 [0.906, 0.938]	0.854 [0.838, 0.867]
Res2Net	0.947 [0.933, 0.959]	0.882 [0.868, 0.895]
Res2Net + FPN	0.951 [0.938, 0.963]	0.896 [0.882, 0.910]
Res2Net + HPFF	0.958 [0.947, 0.969]	0.905 [0.893, 0.918]
Res2Net + HPFF + GCA	0.969 [0.959, 0.979]	0.920 [0.908, 0.931]
Res2Net + HPFF + SA	0.961 [0.949, 0.971]	0.913 [0.902, 0.924]
Res2Net + HPFF + CBAM	0.963 [0.951, 0.973]	0.918 [0.905, 0.929]
Res2Net + HPFF + GCSAM (proposed)	0.977 [0.966,0.985]^a^	0.929 [0.917, 0.938]

^a^The 95% confidence intervals were estimated using 1000 bootstrap resampling iterations.

The first experiment utilized ResNet as the backbone network, achieving a sensitivity of 0.923 at 8 FPs/scan and a CPM value of 0.854. This indicates that while ResNet provides basic feature extraction capabilities, its performance in multi-scale lung nodule detection tasks remains limited. Replacing ResNet with Res2Net improved sensitivity to 0.947 and CPM value to 0.882, demonstrating that Res2Net enhances detection performance through multi-scale feature extraction. Further incorporation of the FPN module increased sensitivity and CPM value to 0.951 and 0.896, respectively, validating the effectiveness of multi-scale feature fusion strategies.

To further enhance the fusion of multi-scale features, the HPFF method was introduced. With HPFF, the model’s sensitivity and CPM value increased to 0.958 and 0.905, respectively. This demonstrates that the HPFF method effectively integrates shallow positional information with deep semantic information in a progressive manner, enabling the model to maintain high sensitivity for both large and small nodules. This stepwise fusion strategy minimizes information loss and improves the detection performance across multiple scales.

To further explore the role of attention mechanisms in feature optimization, Global Channel Attention (GCA) and Spatial Attention (SA) were separately introduced on top of HPFF. The CPM values for Res2Net + HPFF + GCA and Res2Net + HPFF + SA reached 0.920 and 0.913, respectively, showing performance improvements. Notably, GCA outperformed SA, possibly because global channel attention was more effective at highlighting important nodule-related features, particularly in lung nodule detection tasks. For comparison, the commonly used Convolutional Block Attention Module (CBAM)^35^ was integrated, and the result showed that Res2Net + HPFF + CBAM achieved a CPM of 0.918. Although CBAM combined attention across both channel and spatial dimensions, it primarily relied on global pooling and did not fully utilize global context information to capture comprehensive features.

Ultimately, the proposed GCSAM module, by integrating GCA and SA mechanisms, effectively fused global contextual information and improved feature selection, demonstrating superior performance. The proposed method incorporating GCSAM achieved the best performance, with a maximum sensitivity of 0.977 and a CPM value of 0.929. Paired t-test^48^ results demonstrated that the improvement in sensitivity at 8 FPs/scan for the proposed method is statistically significant (p < 0.05), further validating the GCSAM module’s ability to fully leverage global contextual information across channel and spatial dimensions, significantly enhancing the model’s detection performance.

### Effect of nodule size on detection

To evaluate the model’s detection performance across different nodule sizes, we divided the 1186 annotated nodules in the LUNA16 dataset into three categories referred to^45^: small nodules (3–5 mm), medium nodules (5–10 mm), and large nodules (> 10 mm), containing 270, 635, and 281 nodules, respectively. Table 4 presents the detection results of the Baseline model and the Baseline + GCSAM model for nodules of different sizes. The Baseline model uses Res2Net as the foundational module for both the detection network and the false positive reduction network, whereas the Baseline + GCSAM model incorporates the GCSAM by replacing the Res2Net module with the Res2GCSA module.

**Table 4:** Performance comparison of the Baseline and Baseline + GCSAM in detecting lung nodules of different sizes on the LUNA16 dataset.

Methods	Number of detected nodules (sensitivity)			Total number of detected nodules	Sensitivity
	3–5 mm	5–10 mm	> 10 mm		
Baseline	245 (0.907)	615 (0.969)	276 (0.982)	1136	0.958
Baseline + GCSAM (proposed)	257 (0.952)	624 (0.982)	278 (0.989)	1159	0.977

As shown in Table 4, the Baseline network performs well in detecting large nodules, as their more prominent features and shapes are easier to capture. However, its performance on small nodules is relatively limited, detecting only 245 small nodules with a sensitivity of 0.907, resulting in a higher miss rate. By incorporating the GCSAM, the Baseline + GCSAM model significantly improves the detection sensitivity for small nodules to 0.952, increasing the number of detected small nodules to 257. This improvement is attributed to the GCSAM module’s ability to effectively combine global channel and spatial attention mechanisms, allowing the model to adaptively focus on the critical features of small nodules, thereby reducing missed detections. For medium nodules, the detection sensitivity of the Baseline + GCSAM model increases from 0.969 to 0.982, indicating that the inclusion of the GCSAM module also benefits the detection of medium nodules. Overall, the Baseline + GCSAM model detects a total of 1159 nodules, surpassing the 1136 nodules detected by the Baseline model, with particularly notable improvements in small nodule detection.

The results indicate that the GCSAM module excels in enhancing the performance of lung nodule detection, particularly in the detection of small nodules. By optimizing global channel and spatial attention mechanisms, GCSAM effectively reduces the miss rate for small nodules and improves the comprehensiveness and accuracy of detecting medium and large nodules.

**Fig. 8:**
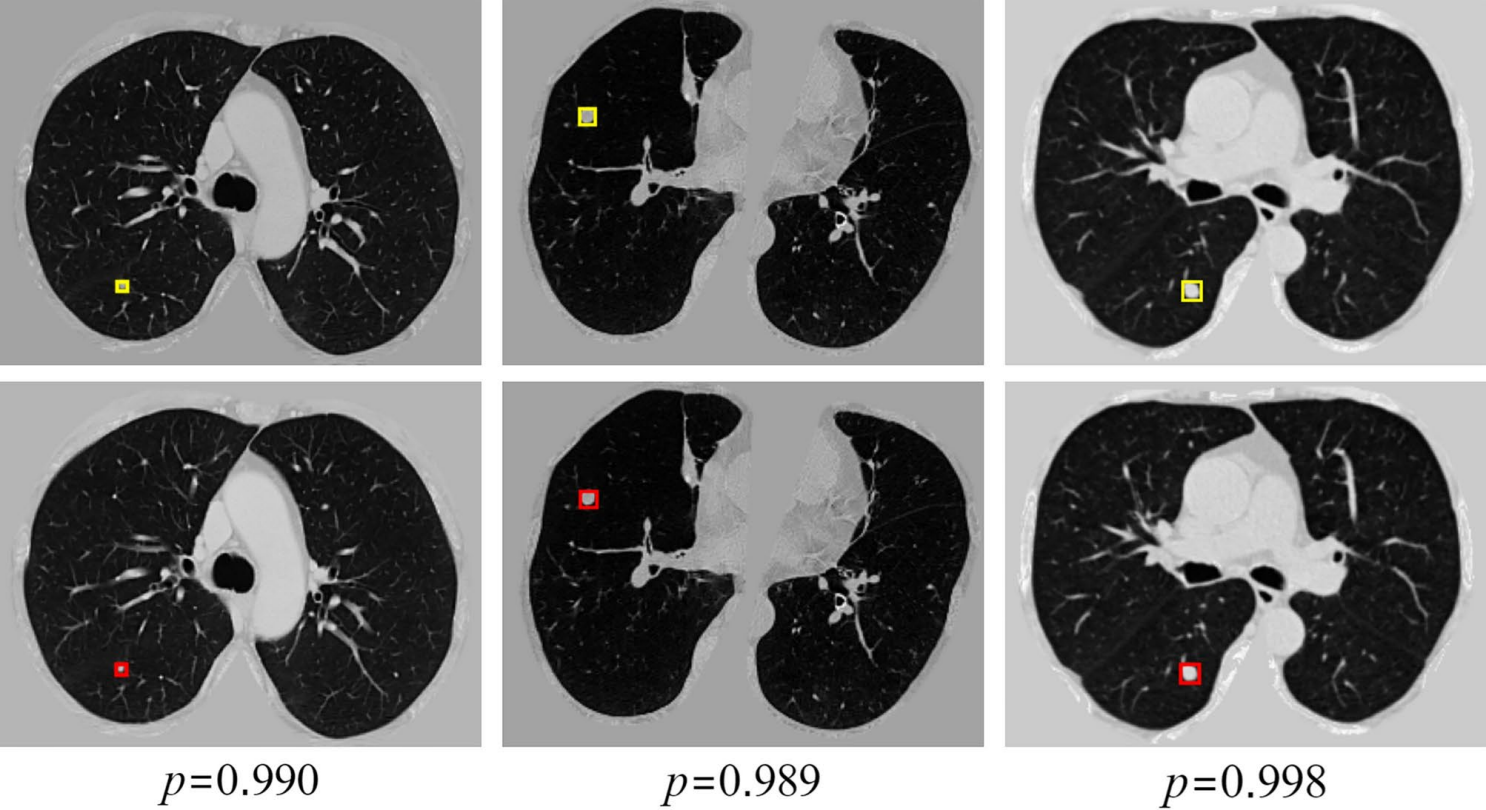
Example of nodule detection of different sizes. The top row shows the ground truth nodules marked with yellow boxes, while the bottom row displays the detected nodules marked with red boxes. The value $P$ represents the confidence score, indicating the probability that the detected region corresponds to a true nodule. Confidence scores above 0.95 are indicative of high detection certainty.

We visualized the results, as shown in Fig. 8. The figure demonstrates that the model can accurately locate and detect nodules of various sizes in CT images. For small nodules, the model also achieves recognition with high confidence.

### Qualitative analysis on detection

**Fig. 9:**
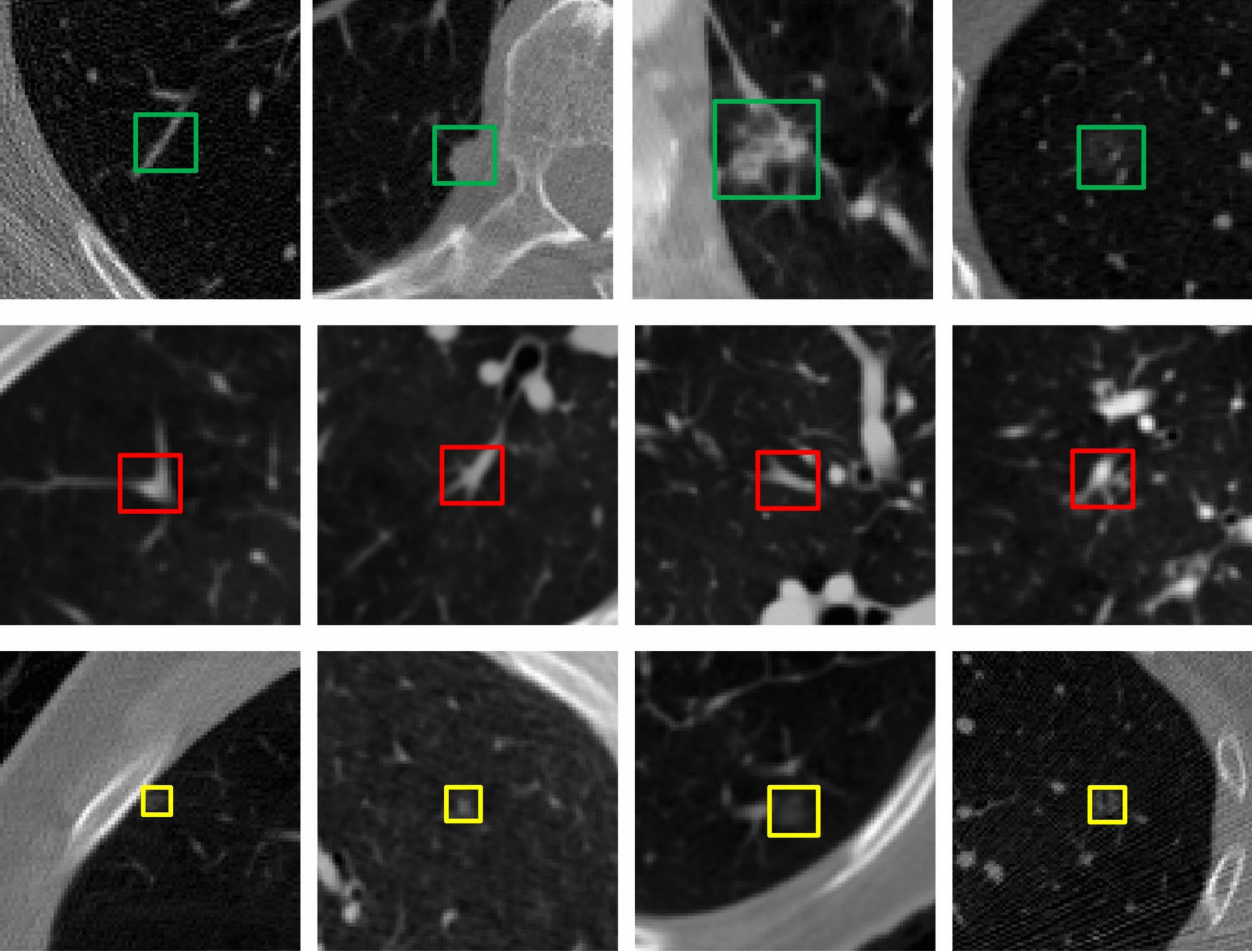
Representative detection results of the proposed method. The first row shows predicted ground truth positive nodules, the second row shows false-positive nodules that were excluded, and the third row shows false-negative nodules that were not detected.

To evaluate the model’s performance in detecting nodules of different morphologies, we conducted a qualitative analysis of the detection results. Figure 9 presents representative outcomes of the model in the lung nodule detection task.

The first row shows nodules successfully detected after incorporating the GCSAM module. Green boxes indicate predicted nodules that align with the ground truth. The results demonstrate that the proposed method exhibits high sensitivity in detecting lung nodules of various morphologies. For instance, complex nodules such as vascular-connected nodules, pleural-attached nodules, irregularly shaped nodules, and ground-glass nodules are accurately identified by the model. This indicates that the inclusion of the GCSAM module, with its combined optimization of global channel and spatial attention mechanisms, enables the model to more effectively capture key features associated with nodules, significantly enhancing its ability to detect complex nodules.

The second row illustrates the performance of the false-positive reduction network. Red boxes mark initially detected false positive nodules, which often possess structural features similar to true nodules. These false positives are successfully filtered out by the FPRNet, demonstrating the model’s robustness in distinguishing true nodules from non-nodule structures in complex backgrounds. However, some false positives remain unfiltered, likely due to the complexity of their features.

The third row shows real nodules that were missed by the model. Yellow boxes indicate ground truth nodules. These missed nodules are typically characterized by extremely small sizes, indistinct features, or high similarity to surrounding tissues. The results suggest that the model still has limitations in handling extremely small or boundary-ambiguous nodules, which may be due to insufficient attention to weak-feature targets, resulting in suboptimal key feature extraction.

These analyses indicate that while the model has made significant progress in detecting complex morphology nodules, there remains room for improvement in reducing missed detections and further lowering the false-positive rate.

## Conclusions

Lung cancer, as a globally high-mortality disease, continues to face challenges such as missed detections of small nodules and high false-positive rates, despite significant advancements in detection and treatment. This paper proposes a two-stage lung nodule detection system that incorporates the GCSAM module and HPFF strategy, significantly enhancing detection sensitivity and accuracy. Experimental results on the LUNA16 dataset demonstrate that the proposed method achieves a CPM value of 0.929 and a sensitivity of 0.977 at 2 FPs/scan, outperforming most existing methods. These results indicate that the proposed model excels in reducing missed detections of small nodules and effectively handling nodules of varying sizes and complex morphologies, highlighting its potential for early screening applications.

Future research could further explore improving the model’s capability to detect extremely small and boundary-ambiguous nodules, which remain common challenges in lung nodule detection. Such nodules often exhibit low contrast and high similarity to surrounding tissues, making feature extraction particularly difficult. Dynamic feature focusing mechanisms could be introduced to enable the model to adaptively concentrate on key features in ambiguous boundary regions. Alternatively, self-supervised learning methods could be employed to enhance feature representation for low-quality data, thereby reducing the miss rate for these nodules. Additionally, to minimize false positives, more efficient feature fusion mechanisms could be developed, or post-processing techniques optimized to better distinguish complex false positive features. These improvements would further enhance the precision and clinical applicability of the model, providing more reliable technological support for early lung cancer screening.

## Data Availability

The data used in this study can be accessed from the LUNA16 official website at https://luna16.grand-challenge.org/Home/.

## References

[1] Siegel, R. L., Miller, K. D., Jemal, A. Cancer statistics, 2015. *CA Cancer J. Clin.* **65** (2015). 10.3322/caac.21254 25559415

[2] Siegel, R. L., Miller, K. D., Wagle, N. S., et al. Cancer statistics, 2023. *CA Cancer J. Clin*. **73** (2023). 10.3322/caac.21763 36633525

[3] Camarlinghi, N. Automatic detection of lung nodules in computed tomography images: training and validation of algorithms using public research databases. *Eur. Phys. J. Plus* **128**, 110 (2013).

[4] Wood, D. E., Kazerooni, E. A., Baum, S. L., et al. Lung cancer screening, version 3.2018, NCCN clinical practice guidelines in oncology. *J. Natl. Compr. Cancer Netw*. **16**, 412–441(2018). 10.6004/jnccn.2018.0020 PMC6476336 29632061

[5] Gould, M. K. et al. Evaluation of individuals with pulmonary nodules: When is it lung cancer?: Diagnosis and management of lung cancer: American College of Chest Physicians evidence-based clinical practice guidelines. *Chest* **143**, e93S-e120S (2013). 23649456 10.1378/chest.12-2351 PMC3749714

[6] Messay, T., Hardie, R. C. & Rogers, S. K. A new computationally efficient CAD system for pulmonary nodule detection in CT imagery. *Med. Image Anal.* **14**, 390–406 (2010). 20346728 10.1016/j.media.2010.02.004

[7] El-Regaily, S. A., Salem, M. A. M., Aziz, M. H. A., et al. Lung nodule segmentation and detection in computed tomography. In *2017 Eighth International Conference on Intelligent Computing and Information Systems (ICICIS)*, 72–78 (IEEE, 2017).

[8] Lu, L. et al. Hybrid detection of lung nodules on CT scan images. *Med. Phys.* **42**, 5042–5054 (2015). 26328955 10.1118/1.4927573

[9] Wang, B. et al. Pulmonary nodule detection in CT images based on shape constraint CV model. *Med. Phys.* **42**, 1241–1254 (2015). 25735280 10.1118/1.4907961

[10] Mei, J. et al. SANet: A slice-aware network for pulmonary nodule detection. *IEEE Trans. Pattern Anal. Mach. Intell.* **44**, 4374–4387 (2021). 10.1109/TPAMI.2021.3065086 33687839

[11] Fu, L. et al. Automatic detection of lung nodules: false positive reduction using convolution neural networks and handcrafted features[C]//Medical Imaging 2017: Computer-Aided Diagnosis. *SPIE* **10134**, 60–67 (2017).

[12] Xie, H. et al. Automated pulmonary nodule detection in CT images using deep convolutional neural networks. *Pattern Recogn.* **85**, 109–119 (2019).

[13] Ren, S. et al. Faster R-CNN: Towards real-time object detection with region proposal networks. *IEEE Trans. Pattern Anal. Mach. Intell.* **39**, 1137–1149 (2016). 27295650 10.1109/TPAMI.2016.2577031

[14] Khosravan, N., Bagci, U. S4ND: Single-shot single-scale lung nodule detection. In *Medical Image Computing and Computer Assisted Intervention–MICCAI 2018: 21st International Conference, Granada, Spain, September 16–20, 2018, Proceedings, Part II 11*, 794–802 (Springer International Publishing, 2018).

[15] Zhu, W., Liu, C., Fan, W., et al. Deeplung: Deep 3d dual path nets for automated pulmonary nodule detection and classification. In *2018 IEEE Winter Conference on Applications of Computer Vision (WACV)*, 673–681 (IEEE, 2018).

[16] Ding, J., Li, A., Hu, Z., Wang, L. Accurate pulmonary nodule detection in computed tomography images using deep convolutional neural networks. In *Proceedings of the International Conference on Medical Image Computing and Computer-Assisted Intervention; Quebec City, QC, Canada. 10–14 September*, 559–567 (2017).

[17] Cao, H. et al. A two-stage convolutional neural networks for lung nodule detection. *IEEE J. Biomed. Health Inform.* **24**, 2006–2015 (2020). 31905154 10.1109/JBHI.2019.2963720

[18] Zhang, Y., Tian, Y., Kong, Y., et al. Residual dense network for image super-resolution. In *Proceedings of the IEEE Conference on Computer Vision and Pattern Recognition*, 2472–2481 (2018).

[19] Tang, S., Yang, M. & Bai, J. Detection of pulmonary nodules based on a multiscale feature 3D U-Net convolutional neural network of transfer learning. *PLoS ONE* **15**, e0235672 (2020). 32845877 10.1371/journal.pone.0235672 PMC7449493

[20] Çiçek Ö, Abdulkadir A, Lienkamp S S, et al. 3D U-Net: learning dense volumetric segmentation from sparse annotation. In *Medical Image Computing and Computer-Assisted Intervention–MICCAI 2016: 19th International Conference, Athens, Greece, October 17–21, 2016, Proceedings, Part II 19*, 424–432 (Springer International Publishing, 2016).

[21] Zheng, S. et al. A lower false positive pulmonary nodule detection approach for early lung cancer screening. *Diagnostics* **12**, 2660 (2022). 36359503 10.3390/diagnostics12112660 PMC9689063

[22] Zhao, Y. et al. Pulmonary nodule detection based on multiscale feature fusion. *Comput. Math. Methods Med.* **2022**, 8903037 (2022). 36590762 10.1155/2022/8903037 PMC9797290

[23] Li, Y., Fan, Y. DeepSEED: 3D squeeze-and-excitation encoder-decoder convolutional neural networks for pulmonary nodule detection. In *2020 IEEE 17th International Symposium on Biomedical Imaging (ISBI)*, 1866–1869 (IEEE, 2020). 10.1109/ISBI45749.2020.9098317 PMC7690332 33250956

[24] Peng, H., Sun, H. & Guo, Y. 3D multi-scale deep convolutional neural networks for pulmonary nodule detection. *PLoS ONE* **16**, e0244406 (2021). 33411741 10.1371/journal.pone.0244406 PMC7790422

[25] Zhao, Y., Wang, J., Wang, X., et al. A new pulmonary nodule detection based on multiscale convolutional neural network with channel and attention mechanism. In *Signal and Information Processing, Networking and Computers: Proceedings of the 8th International Conference on Signal and Information Processing, Networking and Computers (ICSINC)*, 1004–1010 (Springer Nature Singapore, 2022).

[26] Cao, Z. et al. Multi-scale detection of pulmonary nodules by integrating attention mechanism. *Sci. Rep.* **13**, 5517 (2023). 37015969 10.1038/s41598-023-32312-1 PMC10073202

[27] Mkindu, H., Wu, L. & Zhao, Y. Lung nodule detection of CT images based on combining 3D-CNN and squeeze-and-excitation networks. *Multimed. Tools Appl.* **82**, 25747–25760 (2023).

[28] Zhang, H., Peng, Y. & Guo, Y. Pulmonary nodules detection based on multi-scale attention networks. *Sci. Rep.* **12**, 1466 (2022). 35087078 10.1038/s41598-022-05372-y PMC8795451

[29] Hu, J., Shen, L., Sun, G. Squeeze-and-excitation networks. In *Proceedings of the IEEE Conference on Computer Vision and Pattern Recognition*, 7132–7141 (2018).

[30] Tajbakhsh, N. et al. Convolutional neural networks for medical image analysis: Full training or fine tuning?. *IEEE Trans. Med. Imaging* **35**, 1299–1312 (2016). 26978662 10.1109/TMI.2016.2535302

[31] Lin, T. Y., Dollár, P., Girshick, R., et al. Feature pyramid networks for object detection. In *Proceedings of the IEEE Conference on Computer Vision and Pattern Recognition*, 2117–2125 (2017).

[32] Gao, S. H. et al. Res2net: A new multi-scale backbone architecture. *IEEE Trans. Pattern Anal. Mach. Intell.* **43**, 652–662 (2019). 10.1109/TPAMI.2019.2938758 31484108

[33] Girshick, R., Donahue, J., Darrell, T., et al. Rich feature hierarchies for accurate object detection and semantic segmentation. In *Proceedings of the IEEE Conference on Computer Vision and Pattern Recognition*, 580–587 (2014).

[34] Cao, Y., Xu, J., Lin, S., et al. Gcnet: Non-local networks meet squeeze-excitation networks and beyond. In *Proceedings of the IEEE/CVF International Conference on Computer Vision Workshops* (2019).

[35] Woo, S., Park, J., Lee, J. Y., et al. Cbam: Convolutional block attention module. In *Proceedings of the European Conference on Computer Vision (ECCV)*, 3–19 (2018).

[36] Wang, X., Girshick, R., Gupta, A., et al. Non-local neural networks. In *Proceedings of the IEEE Conference on Computer Vision and Pattern Recognition*, 7794–7803 (2018).

[37] Srivastava, N. et al. Dropout: a simple way to prevent neural networks from overfitting. *J. Mach. Learn. Res.* **15**, 1929–1958 (2014).

[38] Setio, A. A. A. et al. Validation, comparison, and combination of algorithms for automatic detection of pulmonary nodules in computed tomography images: the LUNA16 challenge. *Med. Image Anal.* **42**, 1–13 (2017). 28732268 10.1016/j.media.2017.06.015

[39] Armato, S. G. III. et al. The lung image database consortium (LIDC) and image database resource initiative (IDRI): a completed reference database of lung nodules on CT scans. *Med. Phys.* **38**, 915–931 (2011). 21452728 10.1118/1.3528204 PMC3041807

[40] Han, Y. et al. Pulmonary nodules detection assistant platform: An effective computer aided system for early pulmonary nodules detection in physical examination. *Comput. Methods Progr. Biomed.* **217**, 106680 (2022). 10.1016/j.cmpb.2022.106680 35176595

[41] Rothe, R., Guillaumin, M., Van Gool, L. Non-maximum suppression for object detection by passing messages between windows. In *Computer Vision–ACCV 2014: 12th Asian Conference on Computer Vision, Singapore, Singapore, November 1–5, 2014, Revised Selected Papers, Part I 12*, 290–306 (Springer International Publishing, 2015).

[42] Bunch, P. C. et al. A free response approach to the measurement and characterization of radiographic observer performance[C]//Application of optical instrumentation in medicine VI. *SPIE* **127**, 124–135 (1977).

[43] Niemeijer, M. et al. On combining computer-aided detection systems. *IEEE Trans. Med. Imaging* **30**, 215–223 (2010). 20813633 10.1109/TMI.2010.2072789

[44] Zhang, F., Xie, Y., Xia, Y., et al. A pulmonary nodule detection method based on residual learning and dense connection. In *Domain Adaptation and Representation Transfer and Medical Image Learning with Less Labels and Imperfect Data: First MICCAI Workshop, DART 2019, and First International Workshop, MIL3ID 2019, Shenzhen, Held in Conjunction with MICCAI 2019, Shenzhen, China, October 13 and 17, 2019, Proceedings 1*, 72–80 (Springer International Publishing, 2019).

[45] Gu, X. et al. The effect of pulmonary vessel suppression on computerized detection of nodules in chest CT scans. *Med. Phys.* **47**(10), 4917–4927 (2020). 32681587 10.1002/mp.14401

[46] Song, W. et al. A multiscale 3D network for lung nodule detection using flexible nodule modeling. *Med. Phys.* **51**(10), 7356–7368 (2024). 38949577 10.1002/mp.17283

[47] Gerald, B. A brief review of independent, dependent and one sample t-test. *Int. J. Appl. Math. Theor. Phys.* **9**(2), 50–54 (2018).

[48] Xu, M., Fralick, D., Zheng, J. Z., Wang, B. & Changyong, F. The differences and similarities between two-sample t-test and paired t-test. *Shanghai Arch. Psychiatry.* **29**, 184 (2017). 28904516 10.11919/j.issn.1002-0829.217070 PMC5579465

